# Industrially Applicable *De Novo* Lager Yeast Hybrids with a Unique Genomic Architecture: Creation and Characterization

**DOI:** 10.1128/AEM.02434-20

**Published:** 2021-01-15

**Authors:** Zachari Turgeon, Thomas Sierocinski, Cedric A. Brimacombe, Yiqiong Jin, Brittany Goldhawke, Jessica M. Swanson, John I. Husnik, Matthew S. Dahabieh

**Affiliations:** a Renaissance BioScience Corporation, Vancouver, British Columbia, Canada; Nanjing Agricultural University

**Keywords:** *Saccharomyces cerevisiae*, *Saccharomyces pastorianus*, fermentation, hybridization, mating, *Saccharomyces eubayanus*, yeasts

## Abstract

Lager beer is produced by Saccharomyces pastorianus, which is a natural allopolyploid hybrid between Saccharomyces cerevisiae and Saccharomyces eubayanus. Lager strains are classified into two major groups based largely on genomic composition: group I and group II. Group I strains are allotriploid, whereas group II strains are allotetraploid. A lack of phenotypic diversity in commercial lager strains has led to substantial interest in the reconstitution of *de novo* allotetraploid lager strains by hybridization of S. cerevisiae and S. eubayanus strains. Such strategies rely on the hybridization of wild S. eubayanus isolates, which carry unacceptable traits for commercial lager beer such as phenolic off flavors and incomplete utilization of carbohydrates. Using an alternative breeding strategy, we have created *de novo* lager hybrids containing the domesticated S. eubayanus subgenome from an industrial S. pastorianus strain by hybridizing diploid meiotic segregants of this strain to a variety of S. cerevisiae ale strains. Five *de novo* hybrids were isolated which had fermentation characteristics similar to those of prototypical commercial lager strains but with unique phenotypic variation due to the contributions of the S. cerevisiae parents. Genomic analysis of these *de novo* lager hybrids identified novel allotetraploid genomes carrying three copies of the S. cerevisiae genome and one copy of the S. eubayanus genome. Most importantly, these hybrids do not possess the negative traits which result from breeding wild S. eubayanus. The *de novo* lager strains produced using industrial S. pastorianus in this study are immediately suitable for industrial lager beer production.

**IMPORTANCE** All lager beer is produced using two related lager yeast types: group I and group II, which are highly similar, resulting in a lack of strain diversity for lager beer production. To date, approaches for generating new lager yeasts have generated strains possessing undesirable brewing characteristics which render them commercially inviable. We have used an alternative approach that circumvents this issue and created new lager strains that are directly suitable for lager beer production. These novel lager strains also possess a unique genomic architecture, which may lead to a better understanding of industrial yeast hybrids. We propose that strains created using our approach be classified as a third group of lager strains (group III). We anticipate that these novel lager strains will be of great industrial relevance and that this technique will be applicable to the creation of additional novel lager strains that will help broaden the diversity in commercial lager beer strains.

## Abstract (precis)

All lager beer is produced using two related lager yeast types: group I and group II, which are highly similar, resulting in a lack of strain diversity for lager beer production. To date, approaches for generating new lager yeasts have generated strains possessing undesirable brewing characteristics which render them commercially inviable.

## INTRODUCTION

The lager beer yeast Saccharomyces pastorianus is an allopolyploid hybrid between Saccharomyces cerevisiae and the cryotolerant species Saccharomyces eubayanus (1, 2). Individual lager strains are classified into two major groups: group I (Saaz type) or group II (Frohberg type). Group I and group II strains have some differences in their genomic compositions and in fermentation characteristics. Phenotypically, group I strains are generally more cryotolerant than group II strains, whereas group II strains display more robust fermentation rates and yields (3, 4). Importantly, group I strains display some phenotypic variation in maltotriose utilization. Some group I strains have an increased maltotriose consumption rate due to the presence of a high-affinity maltotriose transporter (MTT1p) enabling them to preferentially uptake maltotriose over maltose (5), whereas others have a non-maltotriose-utilizing phenotype (5). Genetically, group I lager strains are characterized by an allotriploid genome composed of a diploid complement of S. eubayanus chromosomes and a haploid set of S. cerevisiae chromosomes (4). Group II lager strains are allotetraploids, with diploid sets of both S. eubayanus and S. cerevisiae chromosomes (4, 6), although various subgenomic aneuploidies have been observed (7). One of the more widely accepted hybridization models for the origins of both lager lineages suggests that an initial hybridization event occurred between a haploid S. cerevisiae ale strain and a diploid S. eubayanus strain (8, 9). This progenitor lager strain then underwent multiple chromosomal recombination events to found the group I lineage (10). Subsequently, a second hybridization event occurred between this progenitor and a second S. cerevisiae ale strain to found the group II lineage (8, 9). On the other hand, recent sequencing data using chromosome-level assemblies suggest that, in fact, both group I and group II lager strains originate from a single hybridization event (7) and that the differences between all known modern group I and II strains are primarily the result of differing evolutionary trajectories following a population bottleneck created by the isolation and propagation of pure cultures during the industrialization of brewer’s yeast (7).

The strong phenotypic and, by extension, genotypic selection of lager yeast since their formation has resulted in a modern reliance on only a few closely related lager yeast strains (from each of the group I and group II lineages) for the entirety of lager beer production globally (11). Not surprisingly, this lack of diversity in available lager strains has created substantial interest in the creation of new lager strains. To date, efforts have focused on trying to create *de novo* “lager-like” alloploid hybrids by selective mating of S. eubayanus to S. cerevisiae ale strains using either non-genetically modified (GM) mating strategies (12–16) or plasmid-assisted mating (17). As the cryotolerance of S. eubayanus is a critical component of the lager yeast phenotype, other cryotolerant species, including S. mikatae, S. kudriavzevii, S. arboricola, and S. uvarum, have also been hybridized to S. cerevisiae in an effort to create novel cryotolerant hybrid strains for both beer and wine fermentations (18). More recently, the inheritance of S. eubayanus mitochondria has been shown to be a major factor influencing cryotolerance of S. cerevisiae × S. eubayanus hybrids (19).

Attempts to create *de novo* lager hybrids using S. eubayanus isolates and S. cerevisiae ale strains have successfully created hybrid strains capable of fermenting beer at lager temperatures (10 to 13°C). However, because of their use of wild undomesticated S. eubayanus, these strains also possess a number of other undesirable attributes that generally preclude resulting strains from the production of prototypical lager beers. One such issue is the inheritance of a phenolic off-flavor (POF)-positive phenotype from wild S. eubayanus, a trait which was lost during the domestication of S. pastorianus and is not present in the S. eubayanus subgenome of S. pastorianus (6, 20, 21). Indeed, all known wild isolates of S. eubayanus are POF positive, as classified by their ability to decarboxylate ferulic acid, a natural component of brewer’s wort, into 4-vinylguaiacol (4VG), thereby giving the resulting beer a clove-like aroma that is considered an off aroma in lager beer (15, 16, 22). Another complication of using wild S. eubayanus isolates in *de novo* lager hybridization is the inability of known S. eubayanus isolates to consume maltotriose (3, 14, 23–25), one of the major sugars in brewer’s wort. Recent studies have demonstrated that maltotriose transporters from the domesticated S. eubayanus subgenome of S. pastorianus have evolved the ability to ferment maltotriose (21, 26), with at least one mechanism appearing to be the development of an ectopic chimeric maltotriose transporter derived from the recombination of individual transporter genes not capable of importing maltotriose (27).

A number of strategies have been, or could be, employed to overcome the limitations of using wild S. eubayanus in *de novo* lager hybridization. Adaptive laboratory evolution (ALE) of either wild S. eubayanus or resulting *de novo* hybrids is a commonly used approach to mitigate undesirable phenotypic traits (22, 27, 28). For example, in order to rid *de novo* lager hybrids (created from wild S. eubayanus parents) of this trait, ALE was used to screen UV-mutagenized S. eubayanus cells for loss of the POF phenotype (22). Alternatively, another study used hybridization to create a triple *de novo* allotetraploid lager hybrid from wild S. eubayanus and two S. cerevisiae strains. The authors then isolated a POF-negative diploid meiotic segregant from the hybrid; however, it is unclear how much (if any) of the S. eubayanus subgenome was present in the resulting diploid segregant (16). Finally, molecular biology tools, such as recombinant DNA and/or CRISPR-Cas9 gene editing could be used for strain bioengineering. Indeed, the POF phenotype has been removed from the S. eubayanus genome by deleting the *PAD1* and *FDC1* genes using CRISPR-Cas9 prior to *de novo* hybridization (29); however, the use of genetic engineering tools results in genetically modified organisms (GMOs) that may not be accepted by consumers and/or industry.

An alternative strategy to create industrially suitable *de novo* lager yeast strains is to directly access the already-domesticated S. eubayanus subgenome by isolating mating-competent meiotic segregants from an allotetraploid S. pastorianus strain. Previous studies have demonstrated that it is possible to isolate mating-competent diploid monosporic clones from S. pastorianus (30–32) and that such spore isolates can be successfully mated together (33, 34). Furthermore, it has also been shown that selectable auxotrophies can be induced using non-GMO methodologies in these S. pastorianus spore isolates (31). Taken together, these techniques provide a feasible non-GM option for rare mating of S. pastorianus spore isolates (containing the domesticated S. eubayanus subgenome and mitochondria) with S. cerevisiae strains to create “domesticated” *de novo* lager hybrids. Interestingly, a similar approach was used to create lager-ale hybrids that had improved stress resistance and fermentation performance at warmer ale temperatures (34). However, to the best of our knowledge, this strategy has not been used previously to develop *de novo* lager yeast strains immediately suitable for industrial lager beer production, i.e., with the ability to ferment at lager temperatures (10 to 13°C) and with suitable sugar utilization, stress tolerance, and aroma production, including a POF-negative phenotype.

In this study, we created and characterized *de novo* lager hybrids, which incorporate the domesticated S. eubayanus subgenome from S. pastorianus, using the aforementioned non-GM approach. We show that such *de novo* hybrid strains have fermentation performance and phenotypic attributes that make them suitable to be applied as industrial lager strains. Importantly, by using different parental strains, we show that we can expand the genetic and phenotypic diversity of the novel lager strains while retaining the lager-like qualities. Lastly, by using whole-genome sequencing of selected *de novo* hybrids, we demonstrate that these strains possess a unique allotetraploid genomic composition of three S. cerevisiae genome complements to one S. eubayanus genome. Taken together, this study provides a novel non-GM tool for the creation of *de novo* lager hybrids that does not necessitate the use of wild or modified S. eubayanus strains. The development and characterization of a set of such hybrids—created by combining the domesticated S. eubayanus subgenome with diverse S. cerevisiae parents—facilitates the expansion of the lager strain clade by introducing a novel group of strains which are suitable for industrial lager beer production.

## RESULTS

### Generation of *de novo* lager hybrids by rare mating of S. cerevisiae and S. pastorianus meiotic segregants.

To develop *de novo* lager yeast hybrids using the domesticated S. eubayanus subgenome of S. pastorianus, we isolated allodiploid meiotic segregants from a group II lager strain and rare mated them to a variety of diploid S. cerevisiae ale strains. In order to do so without the use of antibiotic resistance or other foreign markers, auxotrophic variants of the diploid meiotic segregants from a group II lager strain (trp^−^) and different diploid ale strains (lys^−^ or ura^−^) were generated using non-GMO methods. Auxotrophs were then rare mated, and hybrids were selected on minimal medium to isolate prototrophic hybrids. Colonies from each set of rare matings were subcultured on minimal medium for two generations to ensure a pure culture and then confirmed as tetraploid by measuring DNA content using flow cytometry. Bona fide tetraploid hybrids were then screened for lager-appropriate phenotypic traits in lab-scale fermentations of standard wort (15 degrees Plato [°P]) at 13°C. The top five performing hybrids based on initial criteria of suitable fermentation kinetics, high maltotriose utilization, complete consumption of maltose, and low production of off-aroma compounds (i.e., dimethyl sulfide [DMS], acetaldehyde, diacetyl, and ethyl acetate) were carried forward for further characterization. Hybrids originated from the following crosses of diploid parental strains: (i) RB-1114, RB-10 × RB-253; (ii) RB-1186, RB-10 × RB-8; (iii) RB-2215, RB-24 × RB-7; (iv) RB-2251, RB-24 × RB-48; and (v) RB-2403, RB-40 × RB-48.

### *De novo* lager hybrids exhibit broadened temperature tolerance.

**FIG 1:**
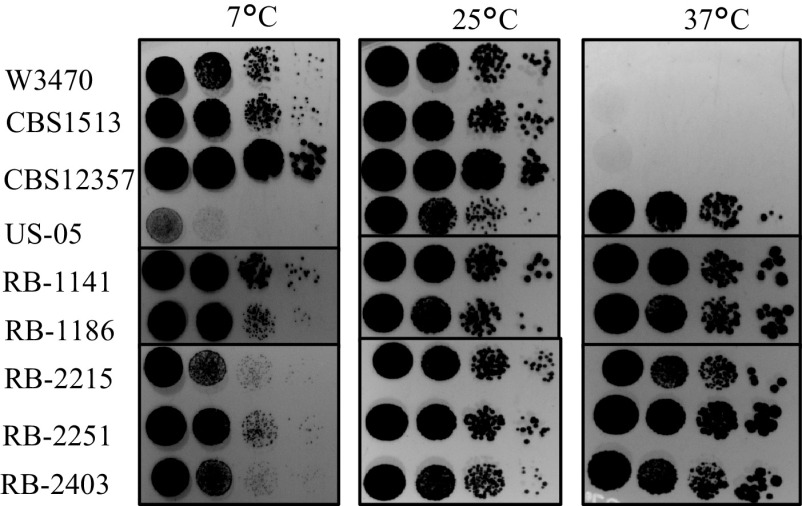
Temperature tolerance of novel hybrids compared to that of group I (CBS1513), group II (W3470), S. eubayanus (CBS12357), and S. cerevisiae (US-05) controls at 7°C, 25°C, and 37°C. Each spot represents a 10-fold serial dilution (10^4^ to 10^1^ cells/spot) on YEG medium.

Temperature tolerance is a key differentiator of S. cerevisiae ale strains and S. pastorianus lager strains, where the former grow well at 37°C but poorly at 7°C, and the latter are incapable of growth at 37°C but grow robustly at 7°C (35). To assess the temperature tolerance phenotype of the novel lager hybrids, we compared the temperature growth profiles of the novel lager hybrids to those of prototypical group I (CBS1513) and group II (W3470) lager strains, S. eubayanus (CBS12357), and a common industrial S. cerevisiae ale strain (US-05). Dilution plating on yeast extract-glucose (YEG) medium at 7°C, 25°C, and 37°C was used to establish the strains’ relative temperature tolerance. As expected based on their established temperature profiles as cryotolerant and cryosensitive strains, respectively (19), S. eubayanus exhibited robust growth at 7°C, while US-05 showed relatively poor growth at 7°C (Fig. 1). Of the novel hybrid lager strains, RB-1141, RB-1186, and RB-2251 had similar growth to that of the S. pastorianus controls (CBS1513 and W3470) at 7°C, with RB-1141 being the most cryotolerant, whereas RB-2215 and RB-2403 had slightly weaker growth at 7°C (Fig. 1). When comparing relative growth at 37°C, neither of the S. pastorianus control strains nor the S. eubayanus strain was able to grow, consistent with previous observations (18, 19, 34); however, all of the novel hybrids were capable of growth at 37°C, similarly to growth of the US-05 S. cerevisiae control (Fig. 1), and all strains had relatively similar growth at the control temperature of 25°C. Taken together, these data indicate that our novel hybrids display heterosis with respect to growth temperature, gaining the ability to grow from 7°C to 37°C, effectively extending their temperature tolerance range.

### *De novo* hybrid lager strains display phenotypic similarities to traditional lager strains in lager beer fermentations.

For any novel lager strain to be suitable for industrial beer production, it must possess suitable traits for lager beer fermentation. Among others, these include complete consumption of maltose and efficient maltotriose utilization at cooler lager fermentation temperatures (10 to 13°C) while producing low levels of acetic acid and other off-aroma compounds. We therefore assessed each hybrid yeast’s performance in lab-scale lager beer brewing compared to that of the group I and group II strains, W3470 and CBS1513, respectively. Briefly, 80-ml fermentations of 15°P wort were inoculated in quadruplicates and incubated isothermally at 13°C. Fermentations were harvested after 14 days, and their sugar profiles were determined by high-performance liquid chromatography (HPLC) analysis. As shown in Table 1, glucose was completely consumed by every strain. Maltose utilization was greater than 98% for all strains with the exception of CBS1513, which only consumed 94.9% of the maltose (3.9 g/liter residual maltose). With respect to maltotriose, all strains exhibited high consumption of maltotriose, consuming between 86.4% to 90.2%. RB-1141 had the overall highest consumption of fermentable sugars (i.e., glucose, maltose, and maltotriose combined), resulting in the highest ethanol concentration (7.4% [vol/vol]), compared with between 7.1% and 7.3% for the other strains. As expected, none of the strains consumed any dextrins (DP4+ sugars), as they all lack the *STA1* gene required for dextrin utilization (36–39). Acetic acid concentration was also measured, and we observed that two of the hybrids, RB-1141 and RB-2403, had elevated levels (0.14 g/liter) compared with those of W3470 (0.10 g/liter) and CBS1513 (0.01 g/liter), while levels in all other hybrids were in between these two lager reference strains.

**TABLE 1:** Sugar utilization of novel hybrids and control group I and group II strains^*a*^

Strain	Sugar utilization (g/liter [% used])					% ethanol produced (vol/vol)
	Glucose	Maltose	Maltotriose	Dextrins	Acetic acid	
Wort	16.1	77.1	26.4	34.7	0.01	0.0
w3470	0 ± 0 (100)	1.3 ± 0.03 (98.3)	3.6 ± 0.04 (86.4)	34.7 ± 0.14	0.10 ± 0.010	7.2 ± 0.03
CBS1513	0 ± 0 (100)	3.9 ± 0.34 (94.9)	3.1 ± 0.07 (88.3)	35.0 ± 0.13	0.01 ± 0.002	7.1 ± 0.01
RB-1141	0 ± 0 (100)	0.8 ± 0.02 (99.0)	2.6 ± 0.10 (90.2)	34.9 ± 0.25	0.14 ± 0.010	7.4 ± 0.05
RB-1186	0 ± 0 (100)	0.9 ± 0.02 (98.8)	3.0 ± 0.05 (88.6)	35.1 ± 0.17	0.06 ± 0.004	7.3 ± 0.02
RB-2215	0 ± 0 (100)	1.2 ± 0.03 (98.5)	3.6 ± 0.01 (86.5)	34.8 ± 0.19	0.09 ± 0.009	7.3 ± 0.03
RB-2251	0 ± 0 (100)	0.8 ± 0.01 (99.0)	3.1 ± 0.06 (88.2)	35.0 ± 0.13	0.09 ± 0.007	7.3 ± 0.03
RB-2403	0 ± 0 (100)	0.9 ± 0.05 (98.8)	3.2 ± 0.06 (87.8)	34.8 ± 0.18	0.14 ± 0.009	7.3 ± 0.05

a Values for unfermented wort are shown, and percent utilization of glucose, maltose, and maltotriose is shown in parentheses next to terminal concentrations.

To analyze fermentation kinetics, diacetyl production, and flocculation in the context of larger scale brewing, we next performed 3-liter lab-scale fermentations. Of note, it was previously observed that both diacetyl (40) and flocculation (41) are better represented in larger volume fermentations, with the increased volume allowing for sampling with minimal disruption to the flocculated yeast at the vessel bottom. Fermentations were carried out isothermally at 13°C in 15°P wort, and samples were taken every 24 h.

**FIG 2:**
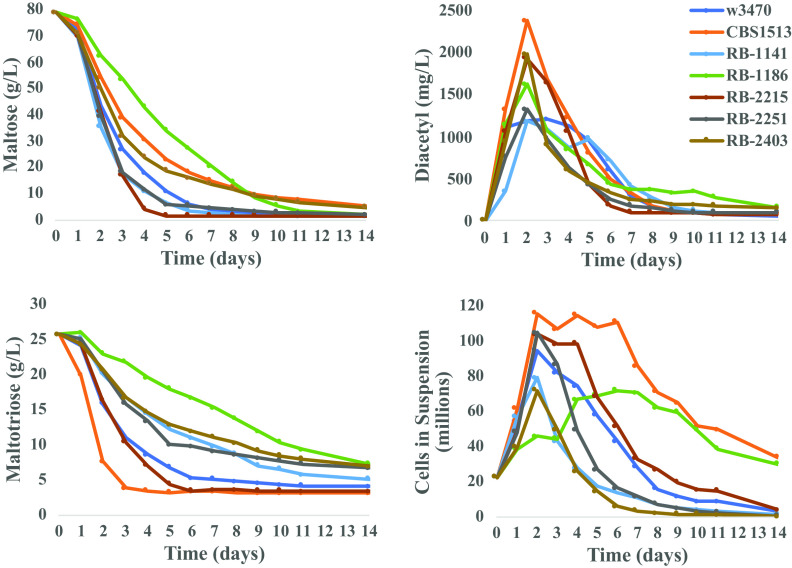
Phenotypic characterization of lager hybrids during beer fermentations performed isothermally at 13°C in 15°P wort in single replicates. Maltose consumption (top left), maltotriose consumption (bottom left), diacetyl production (top right), and cells in suspension (flocculation) (bottom right), shown over a 14-day time course.

Comparing maltose consumption, the best performing strain was RB-2215 (finished on day 5) followed by RB-1141, RB-2251, W3470, RB-2403, CBS1513, and RB-1186, being the slowest, finishing on day 11 (Fig. 2, top left). For maltotriose consumption, the fastest strain was CBS1513 (finishing on day 3), followed by strains RB-2215, W3470, RB-2251, RB-1141, RB-2403, and RB-1186, the slowest, which was still not finished consuming maltotriose after day 14 (Fig. 2). Of note, the ability of the group I control strain CBS1513 to rapidly consume maltotriose was previously observed (5), a finding supported by our results. With respect to diacetyl, we observed that all strains differed in the peak concentration achieved on day 2 as well as the ability to reduce diacetyl concentrations toward the end of the fermentations. The strain with the highest peak diacetyl production was CBS1513 at almost 2,400 mg/liter, followed by RB-2215, RB-2403, RB-1186, RB-2251, W3470, and RB-1141, which had the lowest peak at only 1,190 mg/liter. When considering diacetyl reduction, strain RB-2215 had the greatest reduction capacity followed by RB-2251, RB-2403, CBS1513, W3470, RB-1141, and RB-1186, which had the lowest reduction capacity. Lastly, flocculation throughout the course of the fermentation was monitored by counting total cells in suspension (CIS). All strains started at the same level due to identical inoculation rates. During the initial growth phase (2 to 3 days), a sharp increase in CIS was observed for all strains followed by a drop in CIS (indicative of flocculation), with the exception of RB-1186, which had a more gradual increase in CIS, peaking after day 6. Following the initial peak for CIS, the strain with the fastest flocculation rate was RB-2403, followed by RB-1141, RB-2251, W3470, RB-2215, RB-1186, and CBS1513, which was the slowest to flocculate (Fig. 2, bottom right). Only strains CBS1513 and RB-1186 remained in suspension by day 14 of the fermentation, indicating nonflocculant phenotypes.

Upon completion of fermentation (day 14), both yeast viability and yeast vitality were assessed by methylene blue staining and measuring intracellular glycogen/trehalose levels, respectively. Of note, glycogen is a critical intracellular storage carbohydrate used as a major energy reserve during the initial stages of fermentation and is important for long-term survival during yeast storage (42, 43), and trehalose is an important component of yeast stress tolerance (42, 44), both of which play critical roles in the ability to repitch a yeast strain into subsequent fermentations (45). As CBS1513 and W3470 are both industrial workhorse strains capable of being repitched multiple times, we compared each of the *de novo* hybrids to these strains as indicators of repitching potential. With respect to glycogen content, strain RB-1186 had the highest level followed by strains CBS1513, RB-2403, RB-2251, W3470, RB-1141, and RB-2215 (Table 2). For trehalose, we observed strain CBS1513 with the highest levels followed by W3470, RB-2403, RB-2251, RB-1186, RB-2215, and RB-1141, with the lowest level (Table 2). Viability was determined for each strain on the final day of fermentation (day 14). Strains W3470, CBS1513, RB-2251, and RB-2403 all had very high viability levels (>95%), whereas strains RB-2215, RB-1186, and RB-1141 had lower viability levels between 78.4 and 91.7%.

**TABLE 2:** Measurement of the key vitality indicators as determined by glycogen and trehalose concentration^*a*^

Strain	Conc. (% [wt/wt])		Viability (%)
	Glycogen	Trehalose	
w3470	15.3 ± 0.19	5.68 ± 0.18	96.4 ± 1.04
CBS1513	33.7 ± 0.52	6.57 ± 0.12	98.8 ± 0.30
RB-1141	12.8 ± 0.41	2.27 ± 0.02	78.4 ± 1.64
RB-1186	35.0 ± 0.46	4.35 ± 0.04	87.5 ± 0.22
RB-2215	12.6 ± 0.04	2.83 ± 0.08	91.7 ± 0.98
RB-2251	23.3 ± 0.28	5.29 ± 0.14	97.3 ± 0.35
RB-2403	28.1 ± 0.25	5.48 ± 0.03	98.9 ± 0.29

a Concentration expressed as percent cell weight and cell viability, as measured by methylene blue staining. All samples were taken on the final day of fermentation.

Lastly, the POF phenotype of the *de novo* hybrids was tested by measuring the ability of each strain to decarboxylate ferulic acid *in vitro*, as described previously (46). As expected, W3470 and CBS1513 tested POF negative, whereas S. eubayanus (CBS12357) tested POF positive (see Table S1 in the supplemental material), consistent with previous studies on the POF-positive phenotype of S. eubayanus (16). All of the novel lager hybrids were POF negative, consistent with the POF status of their constituent parent strains.

### Volatile profiling of novel hybrid strains reveals diverse aroma production profiles.

**FIG 3:**
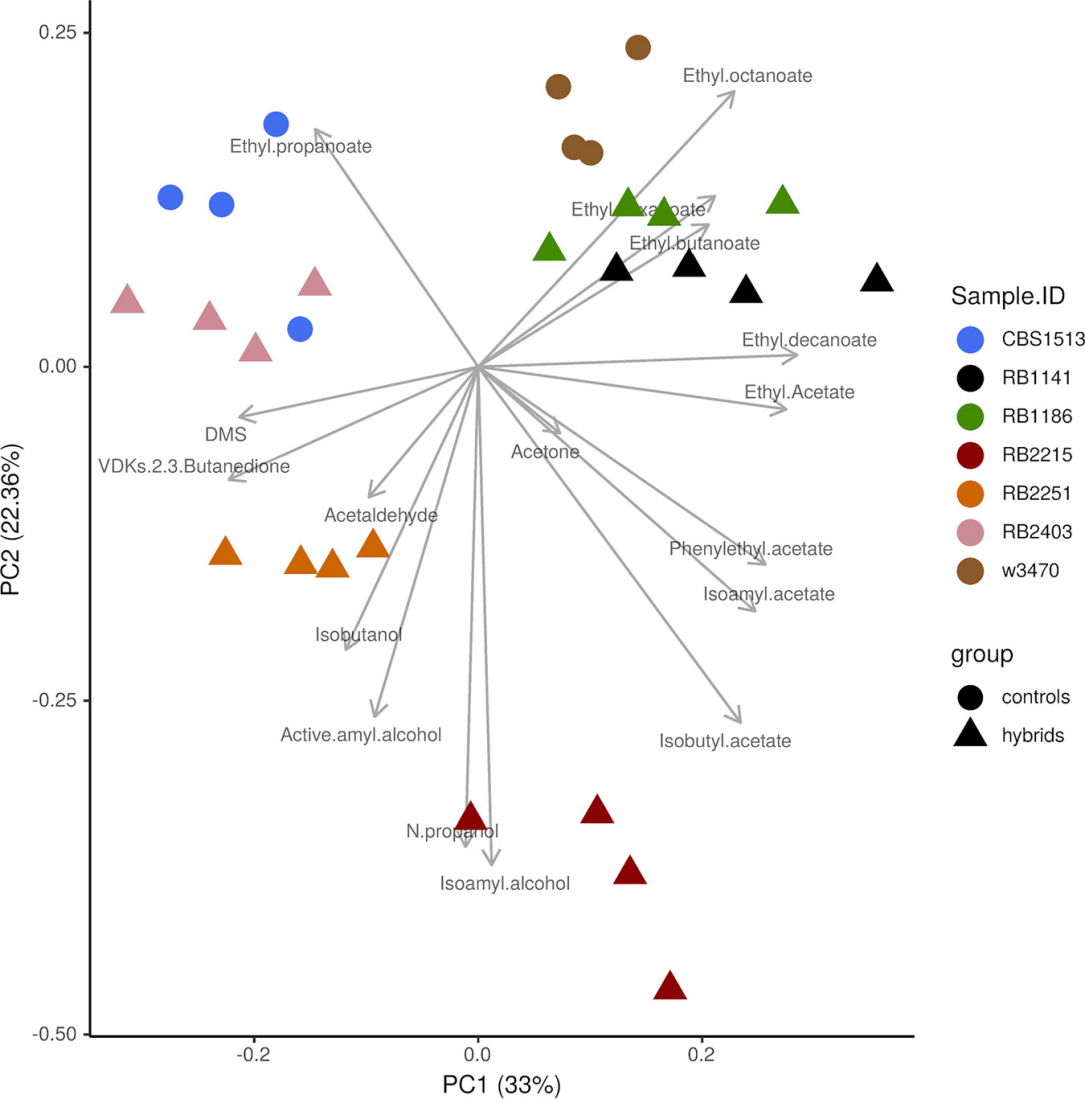
Two-dimensional principal-component analysis (PCA) of volatile compound production by novel lager hybrids and control group I (CBS1513) and group II (W3470) S. pastorianus strains.

**FIG 4:**
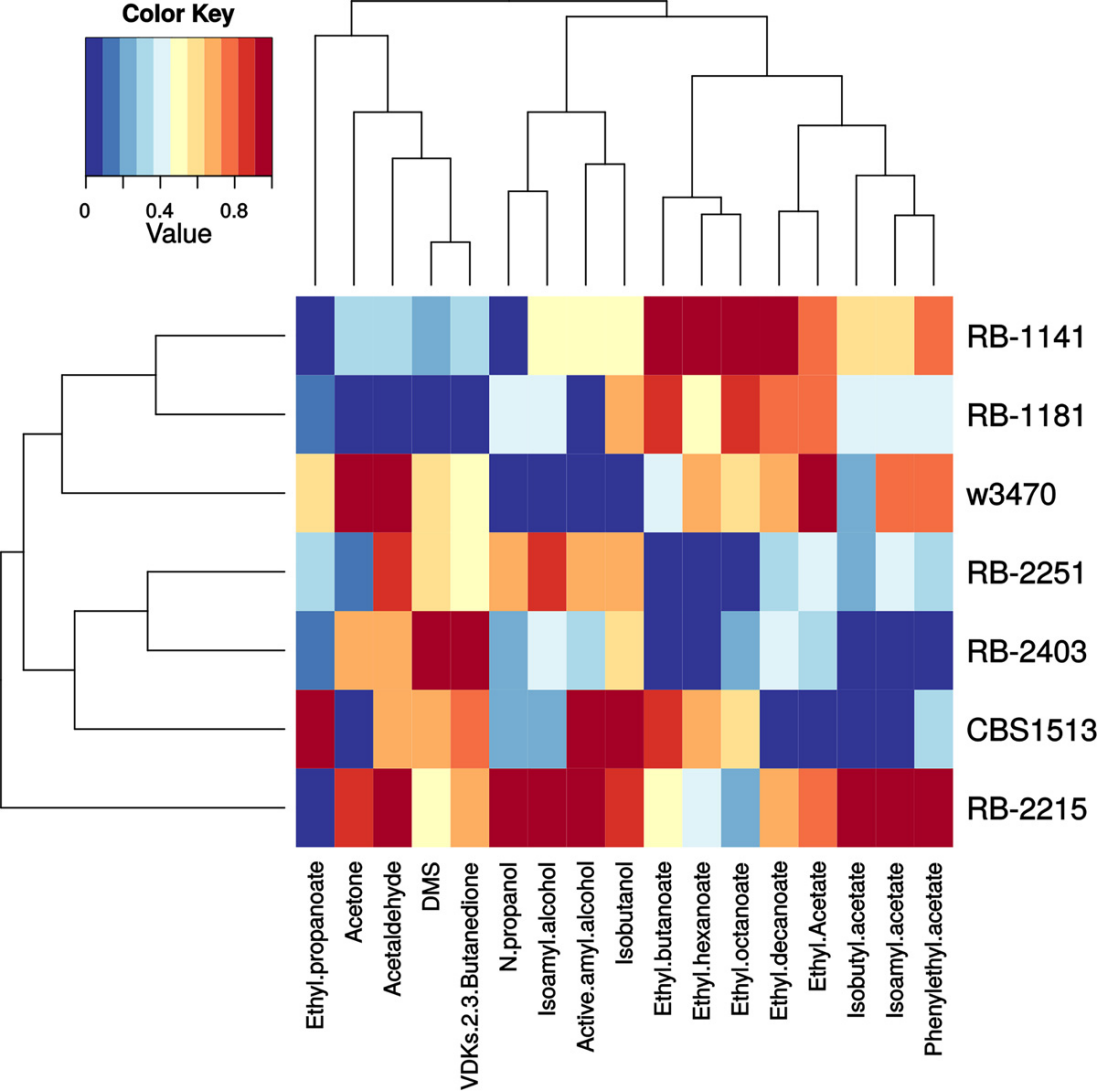
Visual representation of the volatile compound production of novel lager hybrids and control group I (CBS1513) and group II (W3470) lager strains. Colors represent the range of compound concentrations normalized for each strain from 0, the lowest concentration (blue), to 1, the highest concentration (red).

The ability of a lager yeast strain to ferment wort sugars to ethanol at low temperatures while producing acceptable levels of acetic acid and diacetyl and flocculating out by the end of the fermentation are critically important performance characteristics for industrial lager beer production. Equally important, especially for product identity, is the ability of strains to produce a lager-specific volatile profile, with characteristic on aromas and free from off aromas. To assess the fermentation volatile profile of the *de novo* lager hybrids, we measured the volatiles present in finished beer fermentations compared to those present in fermentations conducted with group I and group II strains, CBS1513 and W3470. We used principal-component analysis (PCA) of measured volatile compounds and focused primarily on beer volatile compounds from three major groups of compounds: acetate esters, ethyl esters, and fusel alcohols. Some off aromas were also included as they also make up the main sensory components of beer, e.g., DMS, acetaldehyde, diacetyl, and acetone. As represented in Fig. 3, principal component 1 (PC1) and PC2 carry 33% and 22% of the data set’s total variance, respectively, and each of them significantly segregates strains from each other (analysis of variance for strain versus PC1, *F* = 28.8, *P* = 4.3e−09; strain versus PC2, *F *= 108.6, *P* = 1.1e−14). Strain RB-2403 clustered most closely to the group I CBS1513 strain, whereas strains RB-1141 and RB-1186 clustered closer to the group II strain W3470. This patterning was also observed in the hierarchical clustering of strains as shown in Fig. 4. Strains RB-2251 and RB-2215 clustered separately (mostly according to PC2), indicating they carry the most different, and therefore unique, volatile profiles compared to those of the rest of the hybrids. Only hybrids RB-1141 and RB-1186 were somewhat similar to each other. All other hybrids demonstrated divergent volatile profiles compared to each other.

To further analyze the volatile data and validate the previous PCA analysis, a comparison of specific volatile compounds measured from each strain is shown as a heat map in Fig. 4. Each compound was normalized across all strains on a scale from 0 (lowest measurement) to 1 (highest measurement). Strain RB-2215 had the highest relative production of acetate esters, whereas strain RB-1141 had the highest relative production of ethyl esters. Strain RB-2215 also had the highest production of higher alcohols. With respect to off aromas, strain W3470 had the highest production of acetone and acetaldehyde, and strain RB-2403 had the highest production of dimethyl sulfide (DMS) and diacetyl. In contrast, strain RB-1186 showed the lowest production of acetone, acetaldehyde, DMS, and diacetyl. Taken together, the data shown in Fig. 3 and 4 demonstrate a diversity in volatile compound production between strains across all three major beer volatile compound groups. Compared to control lager strains, the novel lager strains generally produce fewer off aromas overall and exhibit volatile profiles suitable for lager beer production.

### Novel lager hybrids contain both S. cerevisiae and S. eubayanus subgenomes.

Having found that the novel lager hybrids exhibited favorable lager beer-producing phenotypes in a brewing application, we turned our focus to the genomic composition of these hybrids. To characterize the genomic features of these strains and demonstrate the validity of our hybridization method, we used whole-genome sequencing to determine the constitution and origin of each strain’s nuclear and mitochondrial genomes. We generated high-coverage data sets for all five novel hybrids and their parental strains as well as the control group I and group II lager strains, all in their native state and ploidy. Sequencing reads were competitively aligned to a S. cerevisiae and S. eubayanus chimeric reference genomes with a median number of mapped and correctly paired reads of ∼98%, and an average coverage of 124 reads across all sequenced libraries. To identify the origins of each strain, variants (single nucleotide polymorphisms [SNPs] and indels) were called and the read coverage was computed for each genomic position. This allowed the proportions of each subgenome and the mitotype to be characterized and enabled us to confirm ploidy values obtained by flow cytometry, identify possible copy number variants (CNVs)/segmental duplications, and determine the parental origin of the genetic material from each individual novel hybrid (Table 3).

**TABLE 3:** Summary of key genomic and karyotypic data comparing novel hybrids to group I (CBS1513), group II (W3470), S. eubayanus (CBS12357), and S. cerevisiae (US-05) controls

Strain	Ploidy^*a*^	Karyotype (S. cerevisiae to S. eubayanus)	Mitotype	Genome length (Mb)		Chromosome duplications		Chromosome deletions	
				*S. cerevisae*	S. eubayanus	S. cerevisiae	S. eubayanus	S. cerevisiae	S. eubayanus
CBS1513	3N	1:2	S. eubayanus	10.0	11.6				
w3470	4N	2:2	S. eubayanus	11.3	11.0				
US05	4N	4:0	S. cerevisiae	11.4	0.1				
CBS12357	2N	0:2	S. eubayanus	0.5	11.6				
RB-1141	4N	3:1	S. eubayanus	11.5	10.6	VI, VII, VIII, IX, XI	5		3, 9
RB-1186	4N	3:1	S. eubayanus	11.4	10.5	III, VII, VIII, IX, XI, XIV, XVI	5	III	3, 9
RB-2215	4N	3:1	S. cerevisiae	11.6	10.1	I, III, VI, IX, X, XIV	7, 9, 10, 12, 14, 15, 16	IV, V, XI, XII, XV, XVI	3, 11
RB-2251	4N	3:1	S. cerevisiae	11.5	10.1	II, VI, VIII, IX, X, XIV	1, 2, 12, 15		3, 11
RB-2403	4N	3:1	S. cerevisiae	11.5	10.6	II, VI, VIII, IX, X, XI, XIII, XIV	2, 6, 7, 12, 16		3

a Ploidy data were rounded to nearest round number to simplify comparison of strain ploidy and karyotype.

To assess the proportion of S. cerevisiae and S. eubayanus DNA in each strain, we computed the proportion of nucleotides covered in both reference subgenomes (S. cerevisiae and S. eubayanus—both with a length of 12.5 Mb). We then used both proportions to calculate S. eubayanus subgenome/S. cerevisiae subgenome mapping ratios of 0.088 and 23.2 for the control S. cerevisiae (US-05) and S. eubayanus (CBS12357) strains, respectively. For the allopolyploid S. pastorianus lager strains (CBS1513 and W3470), which are known to carry both subgenomes, we observed ratios of 1.16 and 0.97, respectively. Importantly, all of the novel S. pastorianus hybrids exhibited ratios in the range of 0.87 to 0.92, values comparatively and marginally lower than for the controls, indicating they all contain both S. cerevisiae and S. eubayanus subgenomes.

In addition to the nuclear genome, we determined the mitotype of each strain. To do this, we computed the coverage of the S. cerevisiae and S. eubayanus mitochondrial sequences from the sequence alignments with the results summarized in Table 3. Unlike the nuclear genome, mitochondria are inherited from only one parent (47); accordingly, the sequencing analysis showed that the control strain US-05 had S. cerevisiae mitochondria, while the control lager strains CBS1513 and W3470 both had S. eubayanus mitochondria. Interestingly, we observed both mitotypes among the collection of novel hybrids, with two hybrids (RB-1141 and RB-1186) carrying S. eubayanus mitochondria, and three hybrids (RB-2215, RB-2251, and RB-2403) carrying S. cerevisiae mitochondria.

### Whole-genome sequencing unveils unique karyotype for novel lager hybrids.

**FIG 5:**
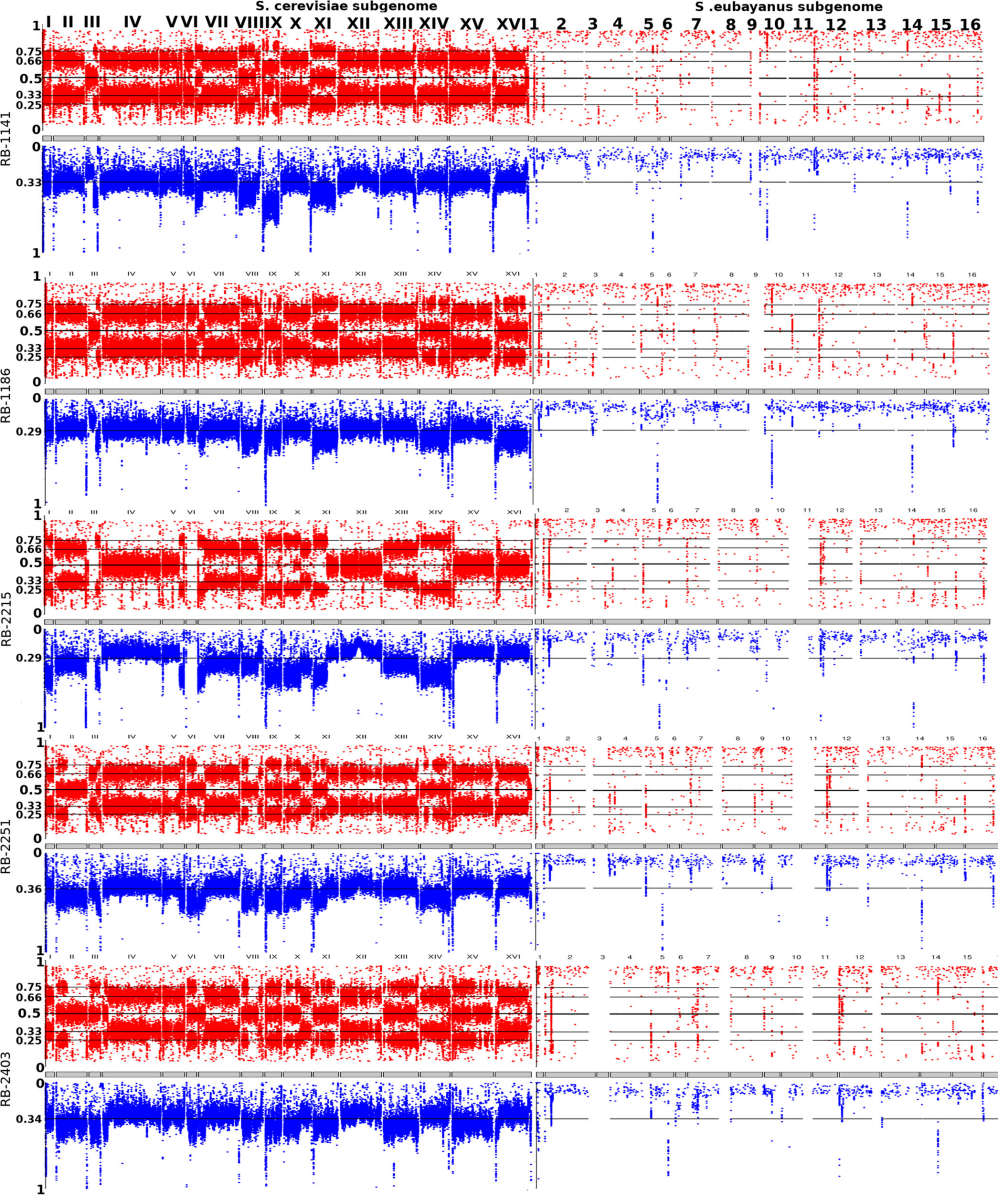
Overview of the SNP frequency (red) and scaled depth of sequencing (0 to 1, in blue) along each subgenome (S. cerevisiae on the left and S. eubayanus on the right) of novel lager hybrids. Strains are RB-1141, RB-1186, RB-2215, RB-2251, and RB-2403.

To more precisely determine the distribution of S. cerevisiae and S. eubayanus DNA in the novel hybrids as well as confirm their parental origins, we characterized the inherited allele frequency across the genome. At each variant (SNP/indel) location, allele frequency was defined by the proportion of reads carrying a given allele. As variants are defined against a reference genome, we picked the most abundant alternate (i.e., nonreference) allele to compute its frequency. This metric characterizes the variety of alleles at the given genomic position but also provides an indirect estimation of copy number/ploidy. For example, a frequency of 0.5 suggests two alleles in a likely diploid region; likewise, 0.33 or 0.66 suggests three possible alleles and thus a possible triploid region, and so on. These data are shown in Fig. 5, with the red dots representing the genome-wide frequencies for each hybrid. Examining the data at the chromosome level, we note that, for the S. cerevisiae part of the subgenome, the frequencies generally followed a uni-, bi-, or trimodal distribution (peaks of 0.5, of 0.33 and 0.66, and of 0.25, 0.5, and 0.75 characterizing a copy number/ploidy of 2, 3, and 4, respectively). The S. eubayanus subgenome displayed mostly frequencies close to 1, indicating the presence of either homozygous or haploid regions. To resolve this ambiguity, as well as other ones inherent to this analysis (e.g., frequencies of 0.5 which could either be diploid or tetraploid), we compared sequencing depth information for every position (Fig. 5). In this analysis, a haploid region is expected to show half of the depth of sequencing of a diploid one, while a tetraploid region is expected to show double the depth of a diploid region, and so on. This additional information allows for the conclusion that only a single copy of the S. eubayanus subgenome is present in most hybrids (as indicated by a depth of sequencing corresponding to one-half that of a diploid S. cerevisiae region in the same hybrid). Overall, the novel hybrids displayed a karyotype showing a majority of triploid regions across the S. cerevisiae subgenome and a single copy of the S. eubayanus subgenome (Fig. 5). We did observe variations to this generalization, with most being located in the S. cerevisiae subgenome. Here, we observed some biallelic/diploid, quadriallelic/tetraploid, and sometimes higher copy number regions (for example, chromosomes III and IX in most strains and X and XI in RB2215, RB2251, and RB2403). Variations were also found in the S. eubayanus subgenome and are documented, along with the S. cerevisiae subgenome ones, in Table 3. Importantly, our results using the above-mentioned approach were corroborated by the measured ploidy state as determined by flow cytometry, similarly to previous studies (48).

To study the parental origins of each hybrid’s genome, we next performed an analysis of SNP inheritance from parents to hybrids. For each hybrid, we first pooled the homozygous private (i.e., specific to an individual parent) SNPs of both corresponding parents. We then intersected that set with the set of variants detected in the hybrid with the idea that since the SNPs are monoallelic in each parent, they can easily be traced in the hybrid’s genomes. As a result of this operation, allele frequency presented here is equivalent to the proportion of sequencing reads mapping to the parental allele. Using this selection, we can compute the frequency of parental alleles and therefore express the proportion alleles inherited from a parent to a hybrid. For example, a frequency of 0.33 for the first parent indicates it contributed one allele to a “triploid” genomic position. Correspondingly, the second parent would have an allele frequency of 0.66 at the same position, indicating a contribution of two of the three alleles present.

**FIG 6:**
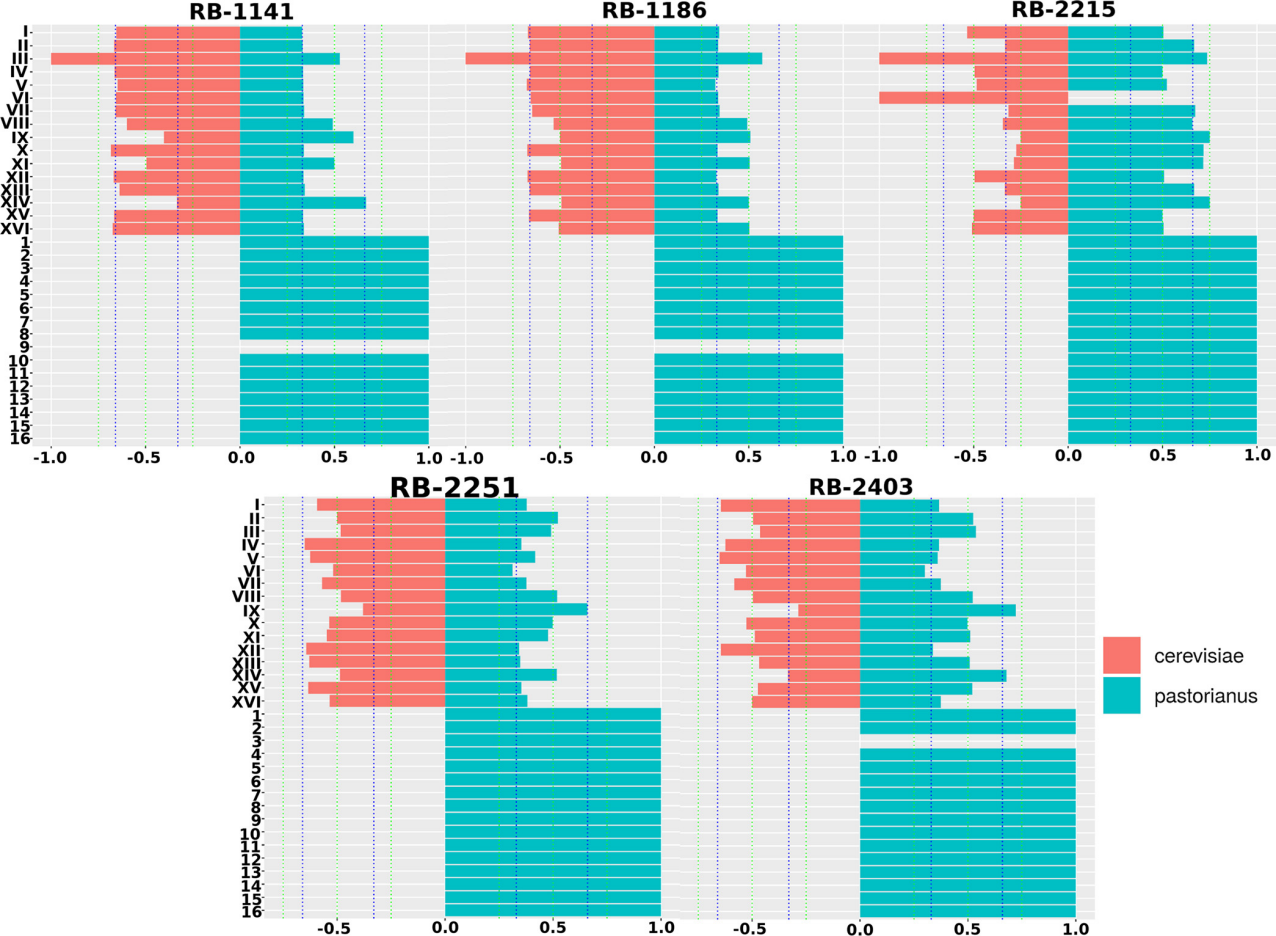
Chromosome-wide parental allele frequency (for S. cerevisiae and S. pastorianus). Vertical lines mark the various ratios, blue as a triploid region mark (at 0.33 and 0.66) and green as diploid/tetraploid region mark (at 0.25, 0.5, and 0.75). Strains are RB-1141, RB-1186, RB-2215, RB-2251, and RB-2403.

Using this methodology, the chromosome-wide median allele frequency of each selected parental allele was calculated for each novel hybrid strain (Fig. 6). Importantly, the novel hybrids generally inherited two copies of their S. cerevisiae subgenome (median allele frequency of 0.6) from their S. cerevisiae parent and one copy from their S. pastorianus parent (median allele frequency of 0.3). This is true for RB-1141, RB-1186, RB-2251, and RB-2403; however, RB-2215 exhibited the converse pattern, with one copy coming from the S. cerevisiae parent and two coming from its S. pastorianus parent (Fig. 6). Of note, due to the copy number variability across the genome and data aggregation, median frequencies can demonstrate some deviation from the expected ratio value. In addition, this expected ratio is computed chromosome wide regardless of possible deletions and solely characterizes the parental inheritance (as opposed to copy number estimation shown previously). For example, even if chromosome 3 is largely deleted in most hybrids, the small section detected in some hybrids originates from the S. pastorianus parent.

## DISCUSSION

The hybrid lager yeast, S. pastorianus, has long been subdivided into group I (Saaz) and group II (Frohberg) strains. Much effort has gone into genotypic and phenotypic characterization of strains from either group. This characterization and the identification and isolation of wild S. eubayanus (2) has allowed researchers to study the *de novo* reconstitution of S. pastorianus from wild S. eubayanus and ale S. cerevisiae strains (12, 14, 16, 17). As discussed previously, such research aims to better understand the evolutionary history of modern lager strains and to increase the phenotypic diversity of lager strains to drive product innovation through novel aromas and flavors in lager beer. In this study, we developed an alternative *de novo* hybridization technique that overcomes previous challenges in strain development by directly breeding the domesticated S. eubayanus subgenome from S. pastorianus lager strains with different S. cerevisiae ale strains. We characterized five *de novo* lager hybrids with favorable lager fermenting phenotypes, and their specific attributes were characterized compared with those of prototypical group I (CBS1513) and group II (W3470) lager strains, as it relates to lager beer production.

The ability of our novel hybrid strains to grow robustly at both 7°C and 37°C indicates a broadened temperature range compared with that of traditional ale or lager strains. This heterotic phenotype may allow our novel hybrids to not only perform at cool lager temperatures but also be suitable in applications as ale strains at warmer temperatures for both fermentation and propagation, although this was outside the scope of the present study. When evaluated in beer fermentations at lager temperatures (13°C), our results indicate that our approach can generate *de novo* lager hybrid strains that are suitable for lager beer production based on their ability to efficiently ferment maltose and maltotriose, reduce diacetyl, flocculate during fermentation, lack the POF trait, and produce volatile profiles which are low in off aromas and have comparable but unique profiles compared with those of prototypical industrial lager strains.

Lastly, genomic characterization of the novel lager hybrid strains revealed a unique genomic composition, where each hybrid had approximately three genomic copies of the S. cerevisiae genome to one copy of the S. eubayanus subgenome. This novel genomic structure has, to the best of our knowledge, never been observed in either natural lager strains or lab-bred *de novo* lager strains. All currently characterized lager strains fall into either the allotriploid group I category, with the genomic composition of 1:2 S. cerevisiae to S. eubayanus, or into the allotetraploid group II category, with the genomic composition being 2:2, which includes *de novo* S. pastorianus strains created from the hybridization of wild S. eubayanus and S. cerevisiae. Our novel lager hybrids fall into what we propose as a group III category, containing a 3:1 S. cerevisiae-to-S. eubayanus genomic structure. We also observed hybrids that contained either an S. cerevisiae or S. eubayanus mitotype, but this did not appear to strongly correlate with increased cryotolerance and did not have an effect on growth above 37°C, indicating additional nuclear genes are important in temperature tolerance in our novel hybrids. This finding was unexpected, as it has been shown that the S. eubayanus mitotype is a critical factor in cryotolerance (47); however, the role of nuclear genes in cryotolerance has also been shown for *Saccharomyces* species (13, 49, 50), and the presence of complementary or compensatory nuclear genes in our hybrids may explain our observations. The parental origin of each chromosome was also traced against each parental genome, and we demonstrate that (with some minor exceptions, most notably, RB-2215) each hybrid inherited two copies of the S. cerevisiae genome from the S. cerevisiae parent, one from the S. pastorianus parent, and of course the lone S. eubayanus copy originating from the S. pastorianus parent.

With respect to fermentation phenotypes, our data suggest that lager hybrids created using our approach can be selected for lager beer fermentation performance while displaying phenotypic diversity across different beer-making parameters. This was expected as all novel hybrids share a similar S. eubayanus subgenomic heritage and partial S. cerevisiae heritage from the S. pastorianus parental strains but highly divergent S. cerevisiae genomic content from the S. cerevisiae ale parent, and hybrids were selected based on similar performance criteria. It is important to note that our results are based on lab-scale fermentations. Furthermore, all hybrids displayed the unique allotetraploid karyotype of 3:1 S. cerevisiae to S. eubayanus for most chromosomes, and the specific parental origin of each chromosome was identified. The phenotypic and genotypic diversity of such hybrids could be further expanded by incorporating S. cerevisiae parents from different geographical or ecological niches with S. pastorianus diploids. Different genomic compositions could also be explored by breeding allodiploid S. pastorianus to haploid S. cerevisiae (2:1 S. cerevisiae-to-S. eubayanus allotriploid) or to diploid S. eubayanus (1:3 S. cerevisiae-to-S. eubayanus allotetraploid). Thus, our hybrids not only show promise as industrial lager beer cultures but may also serve as tools to study the domesticated S. eubayanus subgenome and provide insight into key genomic features critical for lager strain domestication.

Taken together, we have demonstrated that, using our non-GMO breeding approach, we can create novel lager strains with unique and diverse genomes yet possess favorable fermentation performance and volatile profiles for lager beer production. It is important to note that in order to validate our novel lager hybrids at industry scale, follow-up studies will be required in order to determine which of the five hybrids is most suitable for scale-up in a brewery setting. However, our approach does have certain limitations and caveats, such as the S. pastorianus parent can only be derived from group II strains, due to the inability of group I strains to sporulate or produce viable spores. It is also worth noting that it is critically important to undergo a rare mating step to produce higher ploidy strains (tetraploid in this study), as the spore-derived allodiploid S. pastorianus parent strains themselves are poor performers compared to the original parents (data not shown), and it has been suggested that higher ploidy is critical for functional attributes of lager hybrids (15). Previous studies on fertility of allotetraploid *Saccharomyces* hybrids suggest that such strains undergoing meiotic genome reduction are sterile due to mating-type heterozygosity (51–53), but sterility can be overcome by loss of the *MAT* locus (chromosome 3 [Chr3]) from one of the subgenomes (54). Importantly, we observed that all *de novo* hybrids from our study lacked Chr3 from the S. eubayanus subgenome. Since the S. eubayanus subgenome is only inherited from the allodiploid S. pastorianus parent, this suggests that the allodiploid parental strains were hemizygous for the *MAT* locus, enabling the restoration of fertility. This greatly increased the efficiency of rare mating between the allodiploid S. pastorianus and diploid *S cerevisiae* strains and also explains why all hybrids were missing S. eubayanus Chr3 despite having different allodiploid S. pastorianus parental strains, indicating an increase in fertility was critical to forming hybrids using this approach.

In conclusion, we have shown that, using our approach, it is possible to create lager hybrids with a novel genomic composition, which when combined with appropriate selection methods, can generate strains that perform like industrial lager strains yet exhibit unique phenotypic diversity and increased growth at higher temperatures. Furthermore, this approach, when incorporated with a highly divergent S. cerevisiae parental strain, should allow the phenotypic diversity of industrial lager cultures to be vastly expanded and help brewers develop hybrid beer styles or even completely novel beer styles.

## MATERIALS AND METHODS

### Strains, media, and culture conditions.

All strains used in this study are listed in Table 4. Strains were cultured in YEG (1% yeast extract, 2% glucose) or YMM (1% yeast extract, 2% malt extract, 2% maltose) at 21°C, with agitation. For auxotrophic selection, three different media were used, SD+5FOA (1.9 g/liter yeast nitrogen base [YNB] without ammonium sulfate or amino acids but with 2% glucose, 0.5 g/liter 5-fluoroorotic acid [5FOA], and 2% agar), SD+FAA (1.7 g/liter YNB without ammonium sulfate or amino acids but with 5% glucose, 0.74 g/liter complete supplement mixture lacking tryptophan, 10 mg/liter l-tryptophan, 0.5 g/liter 5-fluoroanthranilic acid [FAA], and 2% agar), and SD+αAA (1.7 g/liter YNB without ammonium sulfate or amino acids but with 2% glucose, 30 mg/liter l-lysine, and 20 g/liter dl-2-α-aminoadipate [αAA]). All plates were incubated at 25°C. Strains undergoing sporulation were grown in YEG to late exponential phase, subcultured in SPO medium (0.05% glucose, 0.1% yeast extract, and 1% potassium acetate), and incubated at 21°C for 7 days.

**TABLE 4:** List of strains used in this study

Strain name	Classification	Species	Source
CBS1513	Group I lager	S. pastorianus	NCYC
w3470	Group II lager	S. pastorianus	Commercial
US05	Ale yeast	S. cerevisiae	Commercial
CBS12357	Wild yeast	S. eubayanus	NCYC
RB-1141	Novel hybrid	S. pastorianus	This work
RB-1186	Novel hybrid	S. pastorianus	This work
RB-2215	Novel hybrid	S. pastorianus	This work
RB-2251	Novel hybrid	S. pastorianus	This work
RB-2403	Novel hybrid	S. pastorianus	This work
RB-10	Lager parent	S. pastorianus	Isolate from W3470^*a*^
RB-24	Lager parent	S. pastorianus	Isolate from W3470^*a*^
RB-40	Lager parent	S. pastorianus	Isolate from W3470^*a*^
RB-253	Ale parent	S. cerevisiae	RBSC^*b*^
RB-7	Ale parent	S. cerevisiae	Isolate from NCYC-1113^*a*^
RB-8	Ale parent	S. cerevisiae	Isolate from NCYC-1113^*a*^
RB-48	Ale parent	S. cerevisiae	RBSC^*b*^

a Diploid meiotic segregant isolated from spores of specified strain.

b Diploid strain from RBSC’s culture collection, originally isolated from nature.

### Diploid isolation, auxotroph generation, and rare mating.

Tetraploid parental strains (Table 4) were sporulated, and ascospores were digested in a Zymolyase solution consistent of 5 mg/ml Zymolyase (Nacalai Tesque, Japan) and 1 M sorbitol. Digestion was carried out at 30°C for 4 h. Spores were then resuspended in water and vortexed to facilitate lysis of any remaining vegetative cells. Spore suspensions were then plated out in serial dilutions and incubated at 25°C for 7 days to allow viable spores to form colonies. Cells obtained from smaller colonies were observed microscopically for reduced cell size compared to that of the 4N parental strains (55), at which point their ploidy was determined by flow cytometry. Variants confirmed to possess roughly half of the parental genome content (∼2N) were screened for fermentation characteristics, and those found to inherit similar features as their parental strain were chosen for rare mating. Auxotrophs were obtained as previously described (56–58). Hybridization of auxotrophs was performed by rare mating as previously described with minor modification (59, 60). Successful hybridization was confirmed by measuring DNA content by flow cytometry and cross-checking for loss of resistance to 5FOA, FAA, or αAA.

### DNA content by flow cytometry.

Cells were grown in 5-ml YEG cultures overnight at room temperature on a roller drum. One hundred-microliter samples of cells were taken and washed twice in 1 ml 50 mM sodium citrate (pH 7.2). Cells were then fixed for 2 h in 1 ml 95% ethanol, followed by two washes in 1 ml 50 mM sodium citrate. Cells were then treated with 1 mg/ml RNase A in 50 mM sodium citrate for 2 h at 50°C. SYTOX green nucleic acid stain (Invitrogen) was then added at a final concentration of 1 µM and incubated for 1 h. Samples were analyzed using an Attune NxT flow cytometer (Thermo Fisher) on the fluorescein isothiocyanate (FITC) channel. Ploidy was estimated as comparisons to BY4741 (∼1N), EC1118 (∼2N), VIN7 (∼3N), and w3470 (∼4N) as well as direct comparisons to the parental strains, resultant diploid spores, other beer strains used in breeding, and the resulting tetraploid mating products.

### Wort preparation and fermentation sampling and analysis.

Wort was prepared by dissolving 120 g/liter dry malt extract (CBW Golden Light; Briess, USA) and 60 g/liter brewers crystals (56% maltose corn syrup solids; BrewCraft, USA) in the required amount of water at 90°C. The solution in the kettle (BrewEasy; Blichmann, Lafayette, IN, USA) was brought to a boil for 5 min, and 1.47 g/liter Magnum hops were then added and boiled for 30 min to achieve an international bitterness unit (IBU) of 30. Then, 0.12 g/liter of nutrients (beer nutrient blend; Wyeast, Odell, OR, USA) were added, and the boiling was continued for 10 min.

Laboratory-scale fermentations were carried out according to a standardized system of assessment for the purposes of benchmarking each yeast’s fermentation performance.

Yeast from agar plates were aerobically propagated in 5 ml of YMM broth (1% yeast extract, 2% maltose, 2% malt extract) in culture tubes at 21°C for 24 h with agitation. Cultures were then scaled up to 50 ml of YMM broth in baffled culture flasks and incubated at 21°C for 48 h with agitation. Cells were counted via hemocytometer, pitched into either 80 ml or 3,000 ml of 15°Plato wort at a rate of 1.5 × 10^6^ cells/ml/°Plato, and incubated isothermally at 13°C. Wort was oxygenated by manual agitation; dissolved oxygen levels were not determined. Fermentations were carried out statically in 3.79-liter glass bottles. Fermentation kinetics at the 3,000-ml scale were tracked by daily monitoring for sugar consumption via HPLC analysis, and kinetics at the 80-ml scale were tracked by weight loss.

Initially, each strain was grown to an optical density at 600 nm (OD_600_) of 1.0, and its cell density was determined using a hemocytometer and normalized to its OD_600_ reading. Throughout the course of the fermentation, a 1-ml aliquot was carefully taken from the center of the fermentation vessel so as not to disturb the settled yeast, the sample was diluted, and an OD_600_ reading was obtained. Cells in suspension were calculated by multiplying the OD_600_ by the normalized OD_600_ for each strain. At the end of the primary fermentation, the fermentation vessel contents were stirred in order to suspend all yeast cells uniformly. Samples were taken, and the cell viability was quantified by methylene blue staining and counting with a hemocytometer.

### Quantification of phenolic off flavor by absorbance.

Yeast cultures were streaked onto YEG agar and incubated for 48 h to generate single colonies. A single colony from each strain was inoculated into 2 ml of YEG and 2 ml of YEG plus 0.1 mg/ml of *trans*-ferulic acid (Sigma-Aldrich, Oakville, Canada) and incubated statically for 4 to 7 days at 21°C. Uninoculated controls (medium blank and ferulic acid control) as well as a positive control (RB-65) and negative control (US-05) were also included. After the incubation period, samples were centrifuged and the supernatant was collected in a microcentrifuge tube. Samples were diluted 10-fold (100 μl of sample in 900 μl of reverse osmosis [RO] water), and absorbance readings were taken at 310 nm. Values were corrected for dilution and compared to that for the medium blank versus the reading for ferulic acid control. POF-positive strains will have an observed absorbance similar to that of YEG, and POF-negative strains will be similar to YEG plus *trans*-ferulic acid. The method adopted from that described in reference 46.

### Sugar, organic acid, glycerol, and ethanol analysis by HPLC-RID.

The sugars, organic acids, glycerol, and ethanol of wort and beer samples were quantitated by an HPLC-refractive index detection (RID) system (1260 Infinity II; Agilent, Santa Clara, CA, USA) equipped with a Rezex ROA-organic acid H^+^ (8%) column (300 by 7.8 mm; Phenomenex, Torrance, CA, USA).

Sample preparation and analysis were carried out as previously described (61) with a few modifications. Fermentation samples were centrifuged at 15,000 rpm for 5 min, followed by filtration of supernatant through a 0.45-μm nylon membrane filter. The filtrate obtained was loaded for sugar, organic acid, glycerol, and ethanol analysis using a mobile phase of 0.005 N H_2_SO_4_ at 60°C with a flow rate of 0.5 ml/min for 35 min. Serial standard solutions of compounds of interest were prepared and analyzed to establish standard calibration curves.

### Volatile aromatic compound analysis by HS-GC-MS.

Beer samples were centrifuged at 4,000 rpm for 10 min, and 5 ml of the obtained supernatant was pipetted into a 20-ml headspace (HS) vial sealed by a metal screw cap with a polytetrafluoroethylene (PTFE)/silicone septum for quantitation of acetaldehyde, dimethyl sulfide (DMS), acetone, higher alcohols, and esters by an Agilent gas chromatograph (GC) 7890B coupled with a PAL autosampler (RSI 85; CTC Analytics, Zwingen, Switzerland) and a mass spectrometry (MS) detector (5977B; Agilent, Santa Clara, CA, USA).

Beer samples were incubated at 40°C for 20 min to reach equilibrium, followed by injection of a 1-ml headspace with syringe temperature set at 70°C, inlet temperature at 200°C, and a split ratio of 10. Separation of volatile compounds was carried out with a DB-WAX UI capillary column (60 m by 0.25 mm, 0.25-μm film thickness; Agilent, Santa Clara, CA, USA) and helium as the carrier gas with a constant flow of 1.2 ml/min. The oven program was as follows: held initial temperature at 35°C for 1 min, increased to 120°C by 15°C/min, increased to 180°C by 5°C/min, further increased to 250°C by 20°C/min, and held at 250°C for 5 min. The temperature of the transfer line to MS was set at 250°C. The detection was performed at total ion current (TIC) mode (*m/z* 25 to 250) with electron-impact ionization at 70 eV, ion source temperature at 230°C, and quadrupole temperature at 150°C.

Acetone, DMS, ethyl valerate, ethyl hexanoate, ethyl octanoate, ethyl decanoate, phenethyl acetate (MilliporeSigma, St. Louis, MO, USA), and a premade standard mixture containing acetaldehyde, *N*-propanol, ethyl acetate, isobutanol, isopropyl acetate, ethyl propanoate, active amyl alcohol, isoamyl alcohol, isobutyl acetate, ethyl butanoate, *N*-butyl acetate, and isoamyl acetate (SPEX CertiPrep Inc., Metuchen, NJ, USA) were used to establish standard calibration curves. Ethyl methyl sulfide and 3-octanol were used as the internal standards for quantitation of DMS and the rest of the compounds, respectively.

### Vicinal diketone analysis by UPLC-QDa.

Vicinal diketone (VDK) analysis of beer samples was carried out according to reference 62 with a few modifications. Beer samples were centrifuged at 15,000 rpm for 4 min. The supernatant of 1.5 ml was transferred to a 2-ml screw-cap tube with addition of 27.8 µl 0.2 N H_2_SO_4_ to adjust the pH to 3.5, followed by 90-min heating at 60°C to convert all VDK precursors to VDKs. Subsequently, 2,3-butandione (diacetyl) and 2,3-pentanedione in the heated beer samples were derivatized by *o*-phenylenediamine dihydrochloride (OPDA). The derivatization products (2,3-dimethylquinoxaline and 2-ethyl-3-methyl-quinoxaline) were separated and detected by an ultraperformance liquid chromatography (UPLC)-QDa system (ACQUITY H-Class; Waters, Milford, MA, USA) equipped with a Waters UPLC BEH C_18_ column (2.1 by 50 mm, 1.7 µm). The detailed elution program is shown in Table 5. Probe temperature, capillary voltage, and cone voltage of the QDa were set at 600°C, 800 V, and 12 V, respectively. Positive ions of *m/z* 159.1 and *m/z* 173.1 were monitored for quantitation of diacetyl and 2,3-pentanedione, respectively

**TABLE 5:** Gradient elution program for VDK analysis by UPLC-QDa

Time (min)	Flow (ml/min)	Gradient (%)^*a*^		
		A	B	C
0 (initial)	0.4	40	25	35
2.8	0.4	40	25	35
2.81	0.4	90	0	10
6.3	0.4	90	0	10
6.31	0.4	40	25	35
9.3	0.4	40	25	35

a The gradient elution was conducted using 100% methanol (A), 80:20 10 mM ammonium acetate buffer (pH 5.85)/methanol (B), and 100% water (mobile phase C).

A series of standard solutions of diacetyl and 2,3-pentanedione were prepared, derivatized, and analyzed to establish standard calibration curves.

### Intracellular glycogen and trehalose analysis in yeast cells.

The intracellular glycogen and trehalose contents in yeast cells were analyzed according to the methods used in references 63 and 64, with a few modifications.

**(i) Glycogen.** A homogenous cell suspension of 3 ml was sampled and washed with 0.9% NaCl solution at 4°C. Intracellular glycogen in the washed cell pellet was released by adding 1 ml of 0.25 M Na_2_CO_3_ solution followed by boiling for 2 h. After cooling to room temperature, 0.6 ml of 1 M acetic acid and 2.4 ml of 0.2 M sodium acetate were added to bring the pH to 5.2 for the subsequent glycogen hydrolysis by adding 50 µl of a 26-U/ml amyloglucosidase solution at 57°C for 40 h. The supernatant of the hydrolysate was loaded for glucose quantitation by HPLC-RID with the same method settings as described above.

**(ii) Trehalose.** A homogenous cell suspension of 10 ml was sampled and washed with 0.9% NaCl solution at 4°C. After discarding the supernatant, 10 ml of Milli-Q water was added to resuspend the yeast cells. Once the yeast were fully suspended, they were immediately boiled for 10 min in a water bath to extract trehalose. The supernatant of the extract was collected and analyzed by HPLC-RID for trehalose (see “Sugar, organic acid, glycerol, and ethanol analysis by HPLC-RID”). Trehalose standard solutions were prepared and analyzed for calibration.

Yeast content in a cell suspension (milligram per milliliter) was determined by recovering yeast cells from a 10-ml cell suspension and drying the yeast pellet at 80°C for 3 h. The glycogen content of yeast was calculated as equivalent glucose amount (milligrams) released from glycogen per 100 mg of yeast (dry basis, % [wt/wt]). Trehalose content was presented as trehalose amount (milligrams) extracted from per 100 mg of yeast (dry basis, % [wt/wt]).

### Reference genomes.

Reference genomes of Saccharomyces cerevisiae S288c R64-1-1 (identifier [ID] 15) and *Saccharomyces eubayanus* (ID 41066) (GCA_001298625.1) were merged into a chimeric reference genome.

### Sequencing and raw read alignment.

Genomic DNA was purified using a standard phenol-chloroform-based extraction. DNA was then treated with RNase A for 1 h at 37°C and repurified with an additional phenol-chloroform extraction. Sequencing libraries were prepared using the Nextera DNA Flex library preparation kit, according to the manufacturer’s instructions (Illumina). Libraries were pooled and loaded onto a NextSeq high-output flow cell, generating paired-end 150-bp reads. Raw base call data (bcl) were converted into FastQ format using the bcl2fastq conversion software from Illumina (version 2.19). Library preparation and sequencing were performed at Sequencing and Bioinformatics Consortium at the University of British Columbia (Vancouver, Canada). Raw read quality control was performed in FASTQC (https://www.bioinformatics.babraham.ac.uk/projects/fastqc/). Read alignment to our chimeric reference genome was performed using BWA (65). Duplicated reads were marked using Picard tools (http://broadinstitute.github.io/picard). Base recalibration was performed using Genome Analysis Toolkit (GATK) (66, 67) according to GATK and next-generation sequencing (NGS) data processing workflow (https://gatk.broadinstitute.org/hc/en-us).

### SNP/indel calling and annotation.

SNP/indel variants were called using the GATK software suite and recommendations (66, 67) (https://gatk.broadinstitute.org/hc/en-us). Variant sites were filtered using the following parameters: depth > 40, QD > 2, FS < 60, MQ > 40. Variant annotation was computed using SnpEff (68) and its associated S. cerevisiae R64-1-1v86 and S. eubayanus annotations databases.

### Allele frequency and parental origin.

SNP filtering was performed using bcftools (72). Allele frequency computing and plotting were performed using the R environment, with vcfR (69) and karyoploteR (70) as additional packages.

### Assembly.

For each strain, reads were assembled into scaffolds using ABySS (71), with a median *N*_50_ of 19,666 bases across studied strains. The length of assemblies is reported here as the sum of the length of the distinct scaffolds obtained with ABySS. To compute the length of each subgenome (strain S288C or S. eubayanus), we assembled the reads mapping to the corresponding regions independently and, similarly, reported the cumulative length of distinct scaffolds.

### Data availability.

Raw reads data were deposited in the NCBI Sequence Read Archive under the reference project accession PRJNA665277.

## Supplementary Material

Supplemental file 1
