# Supplementary material for: Industrially Applicable De Novo Lager Yeast Hybrids with a Unique Genomic Architecture: Creation and Characterization
Source: Appl Environ Microbiol. 2021 Jan 15;87(3):e02434-20. doi: 10.1128/AEM.02434-20 (PMC7848916; doi:10.1128/AEM.02434-20)
Supplement: Supplemental file 1 [file AEM.02434-20-s0001.pdf]

Supplemental Material

Sample	Absorbance (310 nm)	POF Result
Media Blank	**4.59**	
Ferulic Acid Control	**10.7**	
Postive control	6.01	Positive
Negative control	11.49	Negative
CBS1513	11.25	Negative
W3470	11.34	Negative
CBS12357	6.33	Positive
RB-1141	10.88	Negative
RB-1186	11.22	Negative
RB-2215	11.11	Negative
RB-2251	11.13	Negative
RB-2403	11.13	Negative

Table S1: Summary of absorbance readings (310 nm) for the detection of trans-ferulic acid for different strains to determine Phenolic-Off Flavour phenotype.

	W3470	CBS1513	RB-1141	RB-1186	RB-2215	RB-2251	RB-2403
Acetaldehyde (ppm)	22 ± 15.1	17 ± 3.6	9 ± 1.9	3 ± 1.2	22 ± 8.2	20 ± 10.8	16 ± 4.3
DMS (ppb)	54 ± 3.5	56 ± 4.2	41 ± 3.0	34 ± 12.6	52 ± 5.2	54 ± 4.8	67 ± 6.2
Acetone (ppm)	1.9 ± 1.54	0.7 ± 0.25	1.0 ± 1.00	0.7 ± 0.05	1.7 ± 2.09	0.9 ± 0.80	1.5 ± 1.68
2,3-butanedione (ppb)	80 ± 8.3	90 ± 12.1	75 ± 7.3	62 ± 5.3	89 ± 5.8	82 ± 6.9	100 ± 6.9
Ethyl acetate (ppm)	40 ± 0.9	19 ± 0.8	34 ± 1.1	35 ± 1.9	36 ± 1.4	28 ± 0.4	26 ± 0.3
Isobutyl acetate (ppb)	83 ± 7.5	64 ± 4.8	113 ± 8.7	94 ± 15.5	144 ± 16.8	80 ± 7.2	68 ± 6.5
Isoamyl acetate (ppb)	1412 ± 158.6	657 ± 86.2	1289 ± 222.1	1045 ± 258.1	1694 ± 361.2	1054 ± 149.3	740 ± 139.3
Phenyletyl acetate (ppb)	835 ± 62.4	592 ± 42.0	876 ± 44.7	646 ± 28.8	1010 ± 50.6	576 ± 54.4	353 ± 62.1
Ethyl propanoate (ppb)	235 ± 27.2	348 ± 44.3	92 ± 6.7	109 ± 5.8	78 ± 9.2	167 ± 2.7	108 ± 3.3
Ethyl butanoate (ppb)	111 ± 8.1	127 ± 7.5	133 ± 7.7	129 ± 8.0	114 ± 9.7	99 ± 11.9	96 ± 10.9
Ethyl hexanoate (ppb)	180 ± 11.5	179 ± 27.7	202 ± 34.1	169 ± 26.5	161 ± 26.1	135 ± 16.8	134 ± 22.9
Ethyl octanoate (ppb)	236 ± 20.1	235 ± 27.3	296 ± 51.8	271 ± 20.1	192 ± 27.4	157 ± 22.3	186 ± 15.0
Ethyl decanoate (ppb)	94 ± 3.5	42 ± 8.3	115 ± 10.7	100 ± 29.3	90 ± 13.8	64 ± 3.4	72 ± 9.7
N-propanol (ppm)	22 ± 1.8	25 ± 3.1	22 ± 1.6	28 ± 1.2	37 ± 1.7	32 ± 2.3	26 ± 2.5
Isobutanol (ppm)	30 ± 0.5	48 ± 2.6	39 ± 0.9	41 ± 2.0	46 ± 2.1	42 ± 1.1	41 ± 0.9
Active amyl alcohol (ppm)	28 ± 0.5	36 ± 1.5	33 ± 0.7	28 ± 0.5	37 ± 0.5	34 ± 0.3	31 ± 0.6
Isoamyl alcohol (ppm)	72 ± 2.2	81 ± 5.9	90 ± 3.0	86 ± 1.5	108 ± 2.5	103 ± 3.5	86 ± 2.6

Table S2: Summary of values obtained by GC-headspace analysis of all volatile compounds measured during beer fermentations. Values are shown plus/minus one standard deviation from quadruplicate fermentations.
